# Potential biomarkers for distinguishing primary from acquired premature ejaculation: A diffusion tensor imaging based network study

**DOI:** 10.3389/fnins.2022.929567

**Published:** 2022-10-19

**Authors:** Jianhuai Chen, Qing Wang, Xinfei Huang, Yan Xu, Ziliang Xiang, Shaowei Liu, Jie Yang, Yun Chen

**Affiliations:** ^1^Department of Andrology, Jiangsu Province Hospital of Chinese Medicine, Affiliated Hospital of Nanjing University of Chinese Medicine, Nanjing, China; ^2^Department of Radiology, Jiangsu Province Hospital of Chinese Medicine, Affiliated Hospital of Nanjing University of Chinese Medicine, Nanjing, China; ^3^Department of Urology, Jiangsu Provincial People’s Hospital, First Affiliated Hospital of Nanjing Medical University, Nanjing, China; ^4^Department of Urology, People’s Hospital of Xinjiang Kizilsu Kirgiz Autonomous Prefecture, Xinjiang, China

**Keywords:** acquired premature ejaculation, diffusion tensor imaging, graph theoretical analysis, segregation, primary premature ejaculation

## Abstract

**Introduction:**

Premature ejaculation (PE) is classified as primary and acquired and may be facilitated by different pathophysiology. Brain plays an important role in PE, however, differences in the central neuropathological mechanisms among subtypes of PE are unknown.

**Materials and methods:**

We acquired diffusion tensor imaging (DTI) data from 44 healthy controls (HC) and 47 PE patients (24 primary PE and 23 acquired PE). Then, the whole-brain white matter (WM) structural networks were constructed and between-group differences of nodal segregative parameters were identified by the method of graph theoretical analysis. Moreover, receiver operating characteristic (ROC) curves were performed to determine the suitability of the altered parameters as potential neuroimaging biomarkers for distinguishing primary PE from acquired PE.

**Results:**

PE patients showed significantly increased clustering coefficient *C*(*i*) in the left inferior frontal gyrus (triangular part) (IFGtriang.L) and increased local efficiency *E*_*loc*_(*i*) in the left precental gyrus (PreCG.L) and IFGtriang.L when compared with HC. Compared to HC, primary PE patients had increased *C*(*i*) and *E*_*loc*_(*i*) in IFGtriang.L and the left amygdala (AMYG.L) while acquired PE patients had increased *C*(*i*) and *E*_*loc*_(*i*) in IFGtriang.L, and decreased *C*(*i*) and *E*_*loc*_(*i*) in AMYG.L. Compared to acquired PE, primary PE patients had increased *C*(*i*) and *E*_*loc*_(*i*) in AMYG.L. Moreover, ROC analysis revealed that PreCG.L, IFGtriang.L and AMYG.L might be helpful for distinguishing different subtypes of PE from HC (PE from HC: sensitivity, 61.70–78.72%; specificity, 56.82–77.27%; primary PE from HC: sensitivity, 66.67–87.50%; specificity, 52.27–77.27%; acquired PE from HC: sensitivity, 34.78–86.96%; specificity, 54.55–100%) while AMYG.L might be helpful for distinguishing primary PE from acquired PE (sensitivity, 83.33–91.70%; specificity, 69.57–73.90%).

**Conclusion:**

These findings improved our understanding of the pathophysiological processes that occurred in patients with ejaculatory dysfunction and suggested that the abnormal segregation of left amygdala might serve as a useful marker to help clinicians distinguish patients with primary PE from those with acquired PE.

## Introduction

Premature ejaculation (PE) is a common male sexual dysfunction characterized by short intravaginal ejaculatory latency time (IELT) and inability to delay ejaculation on all or nearly all vaginal penetrations, as well as negative personal consequences related to PE (Althof et al., 2014; El-Hamd et al., 2019). The population of men with PE is inhomogenous. The most up dated classification by the International Society for Sexual Medicine (ISSM) (Althof et al., 2014) classified PE as primary (prevalence in general population was 2.3–3.2%) and acquired (prevalence in general population was 3.9–4.8%) in 2014 (Serefoglu et al., 2011; Gao et al., 2013). A man would be diagnosed as having primary PE if he experienced PE from the first sexual encounters and remained a problem throughout life. Acquired PE patients often develop PE at some point in their lives after a period of normal ejaculatory function. The classification of primary and acquired PE provides a better perspective of the epidemiology, etiology and treatment of PE.

Although primary and acquired PE patients share similar clinical features, they have different demographic characteristics and etiological factors. Acquired PE is commonly due to psychological or relationship problems and acquired PE patients are usually older, have a higher BMI and a greater incidence of comorbid disease, such as prostatitis, when compared to primary PE (McMahon et al., 2016). ISSM guidelines determine that the off-label daily usage of paroxetine, sertraline, escitalopram and fluoxetine and on-demand treatment with paroxetine and sertraline for treatment of primary and acquired treatment of PE has an evidence level of 1a (Althof et al., 2010). The well-described clinical effects of selective serotonin reuptake inhibitors (SSRIs) suggested that both primary and acquired PE were associated with brain changes, including abnormalities of central neurotransmitter and structure or function (Chen et al., 2021; Liu et al., 2021; Yang et al., 2021). Some brain regions, such as hypothalamus (Gao et al., 2020) and amygdala (Xu et al., 2021), recently received significant attention with the treatment of SSRIs in PE. However, the different central pathological mechanisms between primary and acquired PE are unknown. Therefore, it is thus necessary to search for reliable biological markers to distinguish primary PE from acquired PE in clinical practice.

Premature ejaculation is often accompanied with psychological problems, such as sexual performance anxiety, depression and relationship problems, as well as chronic prostatitis, a risk factor of PE, which may activate the sympathetic nervous system and reduce the ejaculatory threshold as a result of an earlier emission phase of ejaculation (Buvat, 2011; Wang et al., 2022). These psychological factors and central nerve sensitization were considered to be associated with impaired brain function and structure (Chen et al., 2020a; Huang et al., 2021). Previous studies have found disrupted topological organization in the white matter brain network of brain diseases, such as depressive and Alzheimer’s disease (Korgaonkar et al., 2014; Yan et al., 2018; Cao et al., 2020). Until now, only a few studies have explored the topological characteristics of the brain network in male sexual dysfunction using the method of graph theory (Chen et al., 2018a,b, 2020c,^2021^). With respect to PE, our previous studies found disrupted topological architectures in the white matter brain network of patients with primary PE and patients with depression (Chen et al., 2020c,^2021^), as well as functional abnormalities in those with high sympathetic activity (Chen et al., 2020b). In addition, brain functional biomarkers were identified for distinguish patients with PE from those with anejaculation by the method of resting-state functional magnetic resonance imaging (MRI) (Chen et al., 2020e). Therefore, we speculated that white matter brain network analysis might be useful for finding potential biomarkers for distinguishing primary from acquired PE.

Diffusion tensor imaging (DTI) is a useful and non-invasive neuroimaging technology, which can be used to explore the changes in the white matter microstructure involved in the central pathophysiology of both neurological and psychiatric disorders (Atkinson-Clement et al., 2017; Tae et al., 2018). The human brain can be regarded as a highly complex, interconnected network (Dong et al., 2014; Mears and Pollard, 2016; Ji et al., 2019). Graph theory provides a powerful method for describing the topological characteristics of the brain network consisting of nodes (brain areas) and edges (functional or structural connections between brain areas) (Vecchio et al., 2017; Sporns, 2018; Farahani et al., 2019; Hallquist and Hillary, 2019). The methods of DTI technology and graph theory are used to construct the white matter brain network, which has an optimal balance between local specialization (high level of local clustering and local efficiency) and global integration (short path lengths and high global efficiency) for information processing (Watson et al., 2019; Chen et al., 2021). Therefore, the graph theoretical analysis of the brain network based on DTI data may be a useful method to explore the potential neurobiological mechanisms of PE at the whole brain level.

In this study, we explored the topological organization of the white matter brain networks and identified between-group differences in nodal segregative parameters including nodal clustering coefficient and local efficiency. Furthermore, we used a receiver operator characteristic (ROC) analysis to investigate whether brain regions with altered segregative parameters could discriminate between primary PE and acquired PE.

## Materials and methods

### Participants

A total of 24 primary PE patients, 23 acquired PE patients and 44 age- and educational level matched healthy controls (HC) were recruited in this study. The study was approved by the ethical committee of Jiangsu Province Hospital of Chinese Medicine, Affiliated Hospital of Nanjing University of Chinese Medicine. The written informed consents were obtained from all participants. The demographic and clinical characteristics of all participants were presented in Table 1.

**TABLE 1:** Demographic and clinical characteristics of participants.

Characteristics	Primary PE (*n* = 24)	Acquired PE (*n* = 23)	HC (*n* = 44)	*F*/*t*	*P*
Age (years)	29.83 ± 5.33	29.61 ± 5.28	30.52 ± 7.39	0.18	0.83^a^
Educational level (years)	14.92 ± 2.43	14.26 ± 2.28	14.48 ± 1.61	0.65	0.52^a^
IIEF-5 (scores)	22.63 ± 0.71	22.74 ± 0.75	22.80 ± 0.70	0.44	0.65^a^
PEDT (scores)*	15.04 ± 2.61	14.09 ± 2.64	3.11 ± 2.08	265.10	< 0.01^a^
IELT (seconds)*	31.33 ± 18.79	33.43 ± 19.02	484.09 ± 107.02	401.23	< 0.01^a^
Duration of PE	25.50 ± 17.36	20.87 ± 11.98	−	1.06	0.29^b^
**GPSEP**					
Amplitude	1.042 ± 0.69	1.17 ± 1.03	−	–0.52	0.61^b^
Latency	40.08 ± 5.19	40.35 ± 4.53	−	–0.19	0.85^b^
**DNSEP**					
Amplitude	0.88 ± 0.45	1.00 ± 0.85	−	–0.63	0.53^b^
Latency	40.67 ± 4.31	39.83 ± 4.74	−	0.64	0.53^b^
**PSSR**					
Amplitude	32.54 ± 22.15	31.82 ± 24.25	−	0.11	0.92^b^
Latency^#^	1399.38 ± 254.16	1214.57 ± 194.33	−	2.79	< 0.01^b^

PE, premature ejaculation; HC, health controls; IIEF-5, international index of erectile function; PEDT, premature ejaculation diagnostic tool; IELT, intravaginal ejaculation latency time.

GPSEP, glans penis somatosensory evoked potential; DNSEP, dorsal nerve somatosensory evoked potential; PSSR, penile sympathetic skin response. Multigroup comparisons were performed using the one-way analysis of variance (ANOVA) test with *post-hoc* contrasts by least-significant difference (LSD) test.

*Indicated significant differences between PE (both primary and acquired PE) and HC groups.

^#^Indicated significant differences between primary and acquired PE groups.

^a^Indicated *P* was acquired using ANOVA (*F*-values).

^b^Indicated *P* was acquired using two sample *t*-test (*t* values). *P* < 0.05 indicated statistically significant differences.

The diagnoses of PE were based on the medical and sexual history of patients. Furthermore, PE was classified as primary PE and acquired PE based on the sexual history of patients. The diagnoses of PE were made according to the PE Guidelines published by International Society for Sexual Medicine (ISSM) (Althof et al., 2014): (1) ejaculation always or nearly always occurred prior to or within approximately 1 min of vaginal penetration since the first sexual experience and remained throughout life (primary PE) or had a clinically significant and bothersome reduction in IELT (reported by themselves) at some point in the life after a period of normal ejaculatory function (acquired PE); (2) inability to delay ejaculation on all or nearly all vaginal penetrations; (3) negative personal consequences related to PE and/or the avoidance of sexual intimacy.

Inclusion criterias for all participants were as follows: (1) aged from 20 to 45 years; (2) right-handed; (3) educational level >9 years; (4) in a stable heterosexual relationship with the same sexual partner for at least 6 months; (5) had normal sexual desire; (6) had regular weekly sexual intercourse. Additional inclusion criterias for patients were as follows: (1) IIEF-5 scores > 21; (2) PEDT scores ≥ 11; (3) IELT < 1 min. Exclusion criterias for all participants were as follows: (1) serious medical disorders, especially psychiatric, or neurologic disorders; (2) substance or alcohol abuse/dependence; (3) head trauma or loss of consciousness; (4) contraindication to MRI scan.

In addition, the erection and ejaculation of patients were assessed using the International Index of Erectile Function (IIEF-5) (Neijenhuijs et al., 2019), Premature Ejaculation Diagnostic Tool (PEDT) (Tang et al., 2018), IELT and the nerve electrophysiological tests, which included the amplitude and latency of dorsal nerve somatosensory evoked potential (DNSEP), glans penis somatosensory evoked potential (GPSEP), and penile sympathetic skin response (PSSR) (Xia et al., 2013, 2014; Yang B. B. et al., 2018) on the day of MRI scan. MRI data were acquired with a 14-day wash-out period, to rule out the influences of drugs that may affect ejaculation and brain function or structure.

### Image acquisition

Magnetic resonance imaging data of all subjects were obtained on a 3.0 T Siemens Verio scanner (Erlangen, Germany). T1-weighted images were acquired with the following parameters: repetition time (TR) = 1,900 ms, echo time (TE) = 2.48 ms, flip angle = 9°, matrix size = 256 × 256, field of view (FOV) = 250 mm× 250 mm, slices = 176, slice thickness = 1 mm, voxel size = 1 × 1 × 1 mm, acquisition time = 4 min 18 s (Chen et al., 2018a,2020c). DTI data were obtained with the following parameters: TR = 6,600 ms TE = 93 ms, flip angle = 90°, matrix size = 128 × 128, field of view (FOV) = 240 mm× 240 mm, slices = 45, slice thickness = 3 mm, voxel size = 1.9 × 1.9 × 3 mm, gradient values *b* = 0 and 1,000 s/mm^2^, non-linear directions = 30, acquisition time = 3 min 46 s (Chen et al., 2018a,2020c).

### Image preprocessing

**FIGURE 1:**
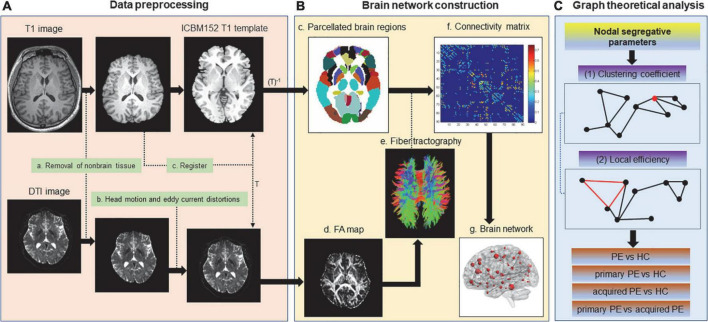
Flowchart for the construction of the white matter anatomical brain network based on DTI data. Non-brain tissues were removed from T1 and DTI images. Eddy current distortions and head motion artifacts were corrected for DTI data. T1 image was co-registered to the non-diffusion image (*b* = 0 image) in DTI native space. The resultant T1 image was registered to the ICBM152 T1 template in the Montreal Neurological Institute (MNI) space, which resulted in a non-linear transformation (T) and an inverse transformation (T^–1^). T^–1^ was applied to the Automated Anatomical Labeling (AAL) template in the MNI space, which resulted in a subject-specific parcellation in the DTI native space. Then diffusion tensors were calculated, FA maps were generated and whole-brain white matter fiber tracking was performed by the probabilistic fiber tracking approach based on DTI data. The weighted connection matrix of 90 AAL regions was created (edges: the average FA of all the voxels along streamlines linking two nodes; nodes: brain regions in AAL template). The weighted white matter anatomical networks were created. Finally, nodal segregative parameters including the clustering coefficient and local efficiency were calculated by the Brain Connectivity Toolbox and comparison of these parameters were performed between groups.

The steps of image processing, brain network construction and graph analysis were illustrated in Figure 1. MRI data were preprocessed using the diffusion toolbox of Functional MRI of the Brain (FMRIB) software Library (FSL)^1^ (Figure 1; Jenkinson et al., 2012). DTI data were preprocessed with the following steps: (1) DICOM data were converted to NIFTI format before processing. (2) The non-brain tissues were split from the images and then brain tissues were extracted. (3) The eddy current distortions and motion artifacts of all diffusion images were corrected by applying the affine transform of diffusion weighted image to the b0 image. (4) The Stejskal and Tanner equation was used to calculate the diffusion tensor matrix. (5) Fractional anisotropy (FA) maps were calculated, which described the distribution of the white matter fiber tracts in the brain.

T1-weighted images were used as the templates of brain areas and were firstly co-registered to the non-diffusion images in the DTI space. The transformed T1 images were segmented into white matter and gray matter, and transformed to the ICBM152 T1 template in the Montreal Neurological Institute (MNI) space, which resulted in a non-linear transformation (T) and an inverse transformation (T^–1^). Then T^–1^ was used to warp the automated anatomical labeling (AAL) templates from the MNI space to the DTI native space.

Finally, Diffusion toolkit^2^ was used reconstruct white matter fiber tracts in the brain using the fiber assignment by continuous tracking (FACT) algorithm. The tractography was terminated when a voxel classified as non-white matter or a voxel with FA value less than 0.2 was reached, or when the turning angle between adjacent voxels was greater than 50°. In addition, the white matter structural connection was adopted when the fiber number was greater than 3, which could remove pseudo connections due to noise effects.

### Network construction

Nodes (brain regions) and edges (structural or functional connections between brain regions) were the two fundamental elements of the network (Rubinov and Sporns, 2010). In the this study, we constructed the white matter structural network using the following procedures: (1) Automated anatomical labeling template (AAL) template (Tzourio-Mazoyer et al., 2002) was subdivided into 90 regions of interest (ROIs) to define the nodes of the brain networks. (2) Two brain regions were considered structurally connected when there were at least three fiber with end points in both regions and the fiber length was greater than 5 mm. (3) The weights of edges were defined by the mean FA values (a very widely used measure of fiber integrity used in DTI studies, ranged from 0 to 1) of the connected fibers between two brain regions. (4) The weighted connection matrix was created and then the weighted brain network was obtained.

### Network analysis

We characterized the nodal segregative function of the brain white matter structural networks using the following two parameters: (1) clustering coefficient: indicating the extent of the local interconnectivity or cliquishness; (2) local efficiency: indicating the ability to transfer local information (Rubinov and Sporns, 2010). These two parameters were often used to detect the ability of brain region for specialized processing to occur within densely interconnected groups of brain regions. High level of segregation suggested an organization of statistical dependencies indicative of segregated neural processing. The nodal clustering coefficient and local efficiency were calculated by the Brain Connectivity Toolbox^3^ (Rubinov and Sporns, 2010).

### Statistical analysis

The Statistical Package for the Social Sciences (SPSS) version 20.0 (IBM, Chicago, IL, USA) was used for statistical analysis. Firstly, the normality and homogeneity of variance were tested by Shapiro–Wilk test and Levene’s test, respectively. Differences of demographic, clinical characteristics and nodal parameters between groups were detected by one-way analysis of variance (ANOVA) test with *post-hoc* contrasts by least-significant difference (LSD) test (two-tailed) when normality and homogeneity of variance assumptions were satisfied. For nodal parameters, false discovery rate (FDR) procedure was applied to address the problem of multiple comparisons. In addition, ROC analysis was constructed to evaluate the accuracy of nodal segregative parameters of different brain regions for discriminating patients with primary PE from those with acquired PE. The significant level was set at *P* < 0.05 (corrected).

## Results

### Demographic and clinical characteristics of all participants

A total of 91 participants were enrolled in the present study (24 primary PE, 23 acquired PE and 44 HC). Demographic and clinical features of all participants were summarized in Table 1. No significant group-differences were found in the age (*F* = 0.18; *P* = 0.83), educational level (*F* = 0.65; *P* = 0.52), duration of PE (*t* = 1.06; *P* = 0.29) and IIEF-5 scores (*F* = 0.44; *P* = 0.65). However, the PEDT scores of the both primary PE and acquired PE groups were significantly higher than those of HC (*F* = 265.10; *P* < 0.05). Moreover, both primary PE and acquired PE patients had decreased IELT when compared with HC (*F* = 401.23; *P* < 0.05). In addition, the results of electrophysiological examinations showed no significant differences between primary and acquired PE groups in the amplitude and latency of GPSEP (*t* = −0.52, *P* = 0.61; *t* = −0.19; *P* = 0.85) and DNSEP (*t* = −0.63, *P* = 0.53; *t* = 0.64; *P* = 0.53), as well as the amplitude of PSSR (*t* = 0.11; *P* = 0.92). However, increased latency of PSSR was found in the group of primary PE group when compared with acquired PE group (*t* = 2.79; *P* < 0.01).

### Nodal segregative parameters between premature ejaculation and healthy controls group

**FIGURE 2:**
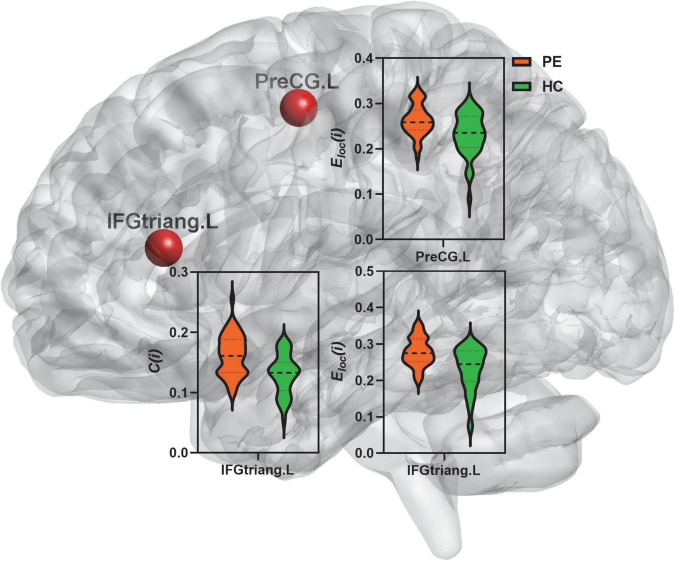
Comparison of nodal segregative parameters between PE and HC groups. PE, premature ejaculation; HC, health controls. IFGtriang.L, left inferior frontal gyrus (triangular part); PreCG.L, left precental gyrus. *C*(*i*), the clustering coefficient of node *i*; *E*_*loc*_(*i*), the local efficiency of node *i*. Comparison was performed using two-sample *t*-test. Bold and thin dashed line dotted lines in the violin plots represented the medians and quartiles of nodal segregative parameters; *P* < 0.05 indicated statistically significant differences.

Compared to the HC group, PE patients exhibited significantly increased clustering coefficient in the left inferior frontal gyrus (triangular part) (*t* = 4.49; *P* < 0.01) and increased local efficiency in the left precentral gyrus (*t* = 3.61; *P* < 0.01) and inferior frontal gyrus (triangular part) (*t* = 4.51; *P* < 0.01) (Table 2 and Figure 2).

**TABLE 2:** Brain regions with abnormal nodal segregative parameters between PE and HC groups.

Segregation	Brain regions	PE (*n* = 47)	HC (*n* = 44)	*t*	*P*
Clustering coefficient	IFGtriang.L	0.16 ± 0.034	0.13 ± 0.033	4.49	<0.01
Local efficiency	PreCG.L	0.26 ± 0.035	0.23 ± 0.046	3.61	<0.01
	IFGtriang.L	0.28 ± 0.039	0.23 ± 0.06	4.51	<0.01

PE, premature ejaculation; HC, health controls; IFGtriang.L, left inferior frontal gyrus (triangular part); PreCG.L, left precental gyrus. Comparisons were performed using two-sample *t*-test. *P* < 0.05 indicated statistically significant differences.

### Nodal segregative parameters among primary, acquired premature ejaculation groups and healthy controls group

#### ANOVA test among three groups

**FIGURE 3:**
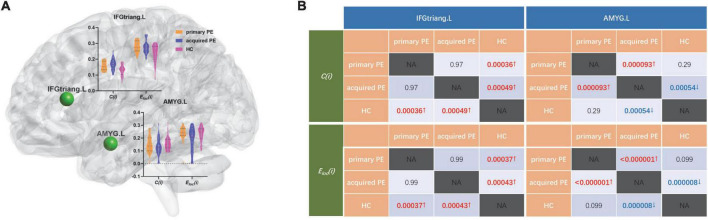
Comparison of nodal segregative parameters between primary PE, acquired PE and HC groups. **(A)** The results of one-way analysis of variance (ANOVA) test among groups, red and black dotted lines in the violin plots represented the medians and quartiles of nodal segregative parameters; **(B)** the results of *post-hoc* contrasts by least-significant difference (LSD) test between groups. PE, premature ejaculation; HC, health controls. IFGtriang.L, left inferior frontal gyrus (triangular part); AMYG.L, left amygdala. *C*(*i*), the clustering coefficient of node *i*; *E*_*loc*_(*i*), the local efficiency of node *i*. NA, not applicable. Red/blue *P* and↑/↓ indicated increased/decreased nodal segregative parameters obtained from the comparisons between primary PE vs. HC; acquired PE vs. HC and primary PE vs. acquired PE. *P* < 0.05 indicated statistically significant differences.

The results of ANOVA analysis showed that there were significant differences in the clustering coefficient and local efficiency of the left inferior frontal gyrus (triangular part) (*F* = 9.98, *P* < 0.01; *F* = 10.04, *P* < 0.01) and amygdala (*F* = 9.50, *P* < 0.01; *F* = 17.59, *P* < 0.01) among the three groups (Table 3 and Figure 3).

**TABLE 3:** Brain regions with abnormal segregative parameters between primary PE, acquired PE and HC groups.

Segregation	Brain regions	Primary PE (*n* = 24)	Acquired PE (*n* = 23)	HC (*n* = 44)	*F*	*P*
Clustering coefficient	IFGtriang.L^a,c^	0.16 ± 0.028	0.16 ± 0.041	0.13 ± 0.033	9.98	<0.01
	AMYG.L^b,c^	0.17 ± 0.063	0.10 ± 0.065	0.16 ± 0.051	9.50	<0.01
Local efficiency	IFGtriang.L^a,c^	0.28 ± 0.038	0.28 ± 0.042	0.23 ± 0.06	10.04	<0.01
	AMYG.L^b,c^	0.26 ± 0.032	0.15 ± 0.094	0.23 ± 0.056	17.59	<0.01

PE, premature ejaculation; HC, health controls; IFGtriang.L, left inferior frontal gyrus (triangular part); AMYG.L, left amygdala. Multigroup comparisons were performed using one-way analysis of variance (ANOVA) test with *post-hoc* contrasts by least-significant difference (LSD) test.

^a^Indicated significant differences between primary PE and HC groups.

^b^Indicated significant differences between primary PE and acquired PE groups.

^c^Indicated significant differences between acquired PE and HC groups. *P* < 0.05 indicated statistically significant differences.

#### *Post-hoc* contrasts between two groups

##### *Primary PE* vs. *HC*

Compared to HC, primary PE patients had increased clustering coefficient and local efficiency in the left inferior frontal gyrus (triangular part) and amygdala (Table 3 and Figure 3).

##### *Primary PE* vs. *acquired PE*

Compared to acquired PE, primary PE patients had increased clustering coefficient and local efficiency in the left amygdala (Table 3 and Figure 3).

##### *Acquired PE* vs. *HC*

Compared to HC, acquired PE patients had increased clustering coefficient and local efficiency in the left inferior frontal gyrus (triangular part), and decreased clustering coefficient and local efficiency in the left amygdala (Table 3 and Figure 3).

### The discriminating value of nodal segregative parameters for premature ejaculation

**FIGURE 4:**
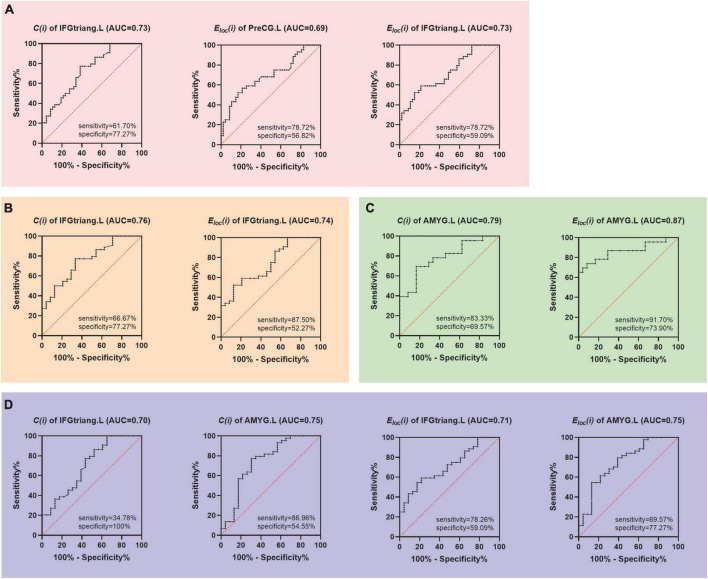
Receiver operator characteristic (ROC) curves based on the abnormal nodal segregative parameters. **(A)** Discrimination PE from HC; **(B)** discrimination primary PE from HC; **(C)** discrimination primary PE from acquired PE; **(D)** discrimination acquired PE from HC. PE, premature ejaculation; HC, health controls. PreCG.L, left precental gyrus; IFGtriang.L, left inferior frontal gyrus (triangular part); AMYG.L, left amygdala. *C*(*i*), the clustering coefficient of node *i*; *E*_*loc*_(*i*), the local efficiency of node *i*. AUC, area under the curve.

Receiver operating characteristic analysis indicated that the clustering coefficient of the left inferior frontal gyrus (triangular part) (AUC = 0.73, *P* < 0.01, sensitivity = 61.70%, specificity = 77.27%; Figure 4A), and the local efficiency of left precental gyrus (AUC = 0.69, *P* < 0.01, sensitivity = 78.72%, specificity = 56.82%; Figure 4A) and inferior frontal gyrus (triangular part) (AUC = 0.73, *P* < 0.01, sensitivity = 78.72%, specificity = 59.09%; Figure 4A) could effectively distinguish PE patients from HC.

The clustering coefficient (AUC = 0.76, *P* < 0.01, sensitivity = 66.67%, specificity = 77.27%; Figure 4B) and local efficiency (AUC = 0.74, *P* < 0.01, sensitivity = 87.50%, specificity = 52.27%; Figure 4B) of the left inferior frontal gyrus (triangular part) were able to distinguish primary PE patients from HC while the clustering coefficient (AUC = 0.79, *P* < 0.01, sensitivity = 83.33%, specificity = 69.57%; Figure 4B) and local efficiency (AUC = 0.87, *P* < 0.01, sensitivity = 91.70%, specificity = 73.90%; Figure 4B) of the left amygdala were able to distinguish primary PE from acquired PE. Moreover, the clustering coefficient and local efficiency of the left inferior frontal gyrus (triangular part) (AUC = 0.70, *P* < 0.01, sensitivity = 34.78%, specificity = 100%; AUC = 0.71, *P* < 0.01, sensitivity = 78.26%, specificity = 59.09%; Figure 4C) and amygdala (AUC = 0.75, *P* < 0.01, sensitivity = 69.57%, specificity = 77.27%; AUC = 0.75, *P* < 0.01, sensitivity = 86.96%, specificity = 54.55%; Figure 4C) all had the ability to distinguish acquired PE from HC (Figure 4).

## Discussion

To find the central neural mechanisms for distinguishing primary PE from acquired PE, we explored the differences of the topological organization of brain white matter structural networks between patients with primary and acquired PE. The main findings were as follows: (1) abnormal segregation of the left inferior frontal gyrus (triangular part) and precentral gyrus might serve as features in the diagnosis of PE. (2) Compared to HC, both primary and acquired PE patients had impaired segregation in the left inferior frontal gyrus (triangular part) and amygdala. (3) Primary and acquired PE patients showed differences in the nodal segregative parameters of the left amygdala. (4) ROC curve analysis indicated that the altered nodal segregation of the left amygdala had high value for distinguishing primary PE from acquired PE. To our knowledge, this study was the first to explore the topologically divergent patterns of the brain white matter networks between primary and acquired PE. These findings might improve our understanding of the neuropathological mechanisms among different subtypes of PE.

Human brain played a pivotal role in the regulation of sexual behavior (Georgiadis and Kringelbach, 2012; Kim et al., 2016; Seok et al., 2016). Sexual pleasure in the appropriate context could lead to excitation and higher arousal, which finally culminated ejaculation and this sexual pleasure disappeared immediately after ejaculation (Georgiadis and Kringelbach, 2012). The brain was found to be strongly correlated with subjective sexual pleasure and the amount of sexual pleasure sufficient to reach ejaculation might be regulated by the balance between sexual inhibitory and excitatory (Georgiadis and Kringelbach, 2012; Zhao et al., 2015; Wang et al., 2017; Jin et al., 2018; Zhang et al., 2022). Normal sexual function was the consequence of the balance between central neural inhibitory and excitatory mechanisms (Rodriguez et al., 2018). In this study, primary PE patients had higher segregation in the frontal-amygdala while acquired PE patients showed higher segregation in the frontal cortex and lower segregation in the amygdala. The imbalance between central neural inhibitory (frontal cortex associated with the symptom of decreased control of ejaculation) and excitatory (amygdala associated with the symptom of rapid ejaculation) might lead to the occurrence of PE. Deactivation of the inferior frontal gyrus was found in individuals during passive viewing of sexual stimuli, which suggested that the inferior frontal gyrus was an inhibitor of sexual arousal (Redouté et al., 2005). In addition, an enhanced activity was found in the left inferior frontal gyrus during the attempted sexual inhibition (Beauregard et al., 2001). Therefore, the left inferior frontal gyrus was considered to be critical for response inhibition/inhibitory control (Swick et al., 2008). Previous functional MRI study demonstrated that PE patients had decreased brain activity in the left inferior frontal gyrus in response to the erotic picture stimuli and at a resting state (Zhang et al., 2017). Activation of left inferior frontal gyrus was found in PE patients in response to inhibition process, which suggested that PE patients had an insufficient control of left inferior frontal gyrus on ejaculation (Yang X. et al., 2018).

As shown in Figure 2 and Table 2, PE patients exhibited increased clustering coefficient in the left inferior frontal gyrus (triangular part) and increased local efficiency in the left precentral gyrus and inferior frontal gyrus (triangular part) when compared with HC. In addition, both primary and acquired PE groups had increased clustering coefficient and local efficiency in the left inferior frontal gyrus (triangular part) (Figure 3 and Table 3). Higher clustering coefficient suggested enhanced interconnections between local brain regions while increased local efficiency indicated higher efficient information transmission among the brain region and its neighbors. Higher level of sexual stimulation experienced by individuals, more requirement for inhibitory control were needed. The increased segregation of left inferior frontal gyrus might demonstrate a compensatory (overactivation) mechanism that required to exert inhibitory control of the ejaculatory response to the erotic stimulus. This finding suggested that patients who were vulnerable to PE had more inhibitory demands during sexual inhibition, which resulted in an increased segregation of left inferior frontal gyrus.

The amygdala was considered to be important for the regulation of male sexual behavior. The sexual pictures and videos could induce significant activations in the amygdala (Mitricheva et al., 2019). Brain activity gradually increased leading up to orgasm and the activated brain regions related to emotional factors included frontal cortical regions and amygdala during orgasm (Wise et al., 2017). Previous study found that the amygdala was found to be a main source of excitatory inputs to the hypothalamus, which played an important role in the ejaculatory response (Yamaguchi et al., 2020). In addition, the amygdala was identified as a sexually dimorphic area, which could increase sexual motivation and the incentive properties of sexual stimulus (Holder and Mong, 2017; Zancan et al., 2018). In contrast to the inhibitory control of sexual behavior, activation of the amygdala was found in individuals who approached sexual stimuli (Stoléru et al., 2012). Moreover, hypersexuality was found in some patients with lesions in the amygdala (Baird et al., 2007). The amygdala was thought to be involved in ejaculation and decreased activation was observed in the amygdala during human male ejaculation, which suggested that amygdala deactivation was correlated with euphoric psychological states (Holstege et al., 2003). For patients with primary PE, smaller bilateral amygdala gray matter volume and impaired functional connectivity of the amygdala were identified in previous neuroimaging study (Geng et al., 2021). The decreased functional connectivity was located between right amygdala and left medial superior frontal gyrus, left insula and right inferior frontal gyrus (Geng et al., 2021; Xu et al., 2021). The functional connectivity between the right amygdala and posterior cingulate cortex and middle temporal cortex showed negative relationships with IELT scores of primary PE patients (Geng et al., 2021). These findings suggested that decreased activation of the amygdala might be related to the impaired functional integration in the perceptual processing of penile inputs and in the awareness of erection in primary PE patients (Geng et al., 2021).

In this study, we also found significant differences in nodal parameters (clustering coefficient and local efficiency) of the left amygdala between primary and acquired PE groups, which suggested pathophysiological differences between primary and acquired PE. Specifically, primary PE patients had increased clustering coefficient and local efficiency in the left amygdala when compared with acquired PE patients (Table 3 and Figure 3). In addition, we found that nodal segregative parameters in the left amygdala had high classification power in discriminating the primary PE from acquired PE. These results were partially consistent with the results of our previous studies, which explored the central neural mechanisms of patients with sexual dysfunction. Our previous study found that lower expression levels of dopamine D2 receptor in the amygdala were observed in rats with non-organic erectile dysfunction (ED), which suggested that the impairment of the dopamine D2 receptor pathway in the amygdala might contribute to the development of non-organic ED (Chen et al., 2018). Moreover, lower connectivity degree and strength was found in psychogenic ED patients and decreased clustering coefficient was found in the amygdala of anejaculation patients in our previous neuroimaging studies, which might contribute to the occurrence of psychogenic ED and anejaculation, respectively (Chen et al., 2018a,2020d).

The increased segregation of the left amygdala in primary PE might reflect facilitated conditioning processes of ejaculatory reflex. Cognitive factors were considered to play an important role in the cascade of sexual arousal responding (Rowland et al., 2013). The physiological signals of sexual responding (genital swelling regulated by brain regions, such as amygdala and insula) could be increased by the cognitive components of the sexual system (such as continuous attention to external sexual stimuli regulated by the prefrontal and anterior cingulate cortex) and the feedback links between cognitive and physical components finally increased the level of sexual arousability (Rowland et al., 2013). PE patients, who experienced long term of difficulties in delaying ejaculation, might have sexual situations with negative expectations (high negative affect and low positive affect) about their sexual performance, which induced an attentional shift toward cues associated with failure anxiety of sexual performance (Dewitte et al., 2021). All these might induce abnormality in the prefrontal and subcortical regions of the brain in PE patients. In addition, the strong evaluative focus on their bodily signals might distract them from erotic cues, which might lead to the occurrence of PE (Stephenson et al., 2018). The prefrontal cortex plays an important role in numerous cognitive functions including regulating arousal and attention (Wegrzyn et al., 2017). Therefore, active-attention (conscious attention focused on a particular intentional goal to achieve or a problem to solve, etc.), which was involved in the voluntary processing of relevant events, was needed to identify the presence of adequate sexual stimuli and to explore whether the current sexual situation was satisfying for eliciting ejaculation (Stephenson et al., 2018). Deficits in the active-attention might lead to an inability to sustain attention on external stimulus, such as sexual stimulation during sexual behavior. Our results suggested the decreased segregation of the left amygdala as the neural underpinning of acquired PE, which might serve as a compensatory mechanism to decrease active-attention to sexual stimulation and promote effective ejaculatory regulation. The abnormal segregation of left amygdala might serve as a useful marker to help clinicians distinguish patients with primary PE from those with acquired PE.

In addition, there were several limitations in this study. Firstly, the small sample size might limit the generalizability of findings in this study. Secondly, the diagnostic criteria for PE were mostly based on patients’ subjective symptoms and lack sensitivity and specificity, which might result in subjective bias. Based on the findings in this study, the technique of neuroimaging might be a useful method for the auxiliary diagnosis of PE, especially patients with higher sympathetic excitability or abnormal emotion or cognition (such as depression and anxiety, as well as impaired attention), which could be identified by structural and functional MRI. Finally, the causality between PE and abnormalities of brain could not be determined due to the cross-sectional study design. Therefore, further longitudinal studies with larger sample size were needed to explore the potential biomarkers to help clinicians distinguish patients with primary PE from those with acquired PE based on neuroimaging data.

## Conclusion

The present study indicated the impaired segregation of the left inferior frontal gyrus (triangular part) and amygdala as the vital neural underpinnings for PE. Primary PE patients developed a compensatory neuro-mechanism with higher segregation in the frontal-amygdala while acquired PE patients developed a compensatory neuro-mechanism with higher segregation in the frontal cortex and lower segregation in the amygdala. The abnormalities in the left inferior frontal gyrus (triangular part) might reflect overlapping pathophysiological processes in primary and acquired PE. In addition, the abnormalities in the amygdala could potentially provide potential biomarkers that would aid in differentiating between primary and acquired PE.

## Data availability statement

The raw data supporting the conclusions of this article will be made available by the authors, without undue reservation.

## Ethics statement

The studies involving human participants were reviewed and approved by the Ethical Committee of Jiangsu Province Hospital of Chinese Medicine, Affiliated Hospital of Nanjing University of Chinese Medicine. The patients/participants provided their written informed consent to participate in this study.

## Author contributions

JC, JY, and YC designed the experiments. JC, QW, XH, YX, ZX, SL, JY, and YC contributed to clinical data collection and assessment. JC, QW, XH, YX, and ZX analyzed the results. JC, QW, and XH wrote the manuscript. All authors have read and approved the final manuscript.

## Footnotes

1
http://www.fmrib.ox.ac.uk/fsl/fdt/index.html

2
http://trackvis.org/dtk/

3
https://www.nitrc.org/projects/bct/
